# Antioxidant, Anti-Inflammatory, Anti-Diabetic, and Pro-Osteogenic Activities of Polyphenols for the Treatment of Two Different Chronic Diseases: Type 2 Diabetes Mellitus and Osteoporosis

**DOI:** 10.3390/biom14070836

**Published:** 2024-07-11

**Authors:** Emanuele-Salvatore Scarpa, Antonella Antonelli, Giancarlo Balercia, Sofia Sabatelli, Filippo Maggi, Giovanni Caprioli, Gilberta Giacchetti, Matteo Micucci

**Affiliations:** 1 R&D Department, Mivell S.r.l.s., 61032 Fano, Italy; 2 Department of Biomolecular Sciences, University of Urbino Carlo Bo, 61029 Urbino, Italy; antonella.antonelli@uniurb.it (A.A.); matteo.micucci@uniurb.it (M.M.); 3 Division of Endocrinology, Department of Clinical and Molecular Sciences, Università Politecnica delle Marche, 60126 Ancona, Italy; giancarlo.balercia@ospedaliriuniti.marche.it; 4 Clinic of Endocrinology and Metabolic Diseases, Department of Clinical and Molecular Sciences, Università Politecnica delle Marche, 60126 Ancona, Italy; s.sabatelli@pm.univpm.it (S.S.); gilberta.giacchetti@ospedaliriuniti.marche.it (G.G.); 5 Chemistry Interdisciplinary Project (CHIP) Research Center, School of Pharmacy, University of Camerino, 62032 Camerino, Italy; filippo.maggi@unicam.it (F.M.); giovanni.caprioli@unicam.it (G.C.)

**Keywords:** polyphenols, type 2 diabetes mellitus, osteoporosis, anti-diabetic, pro-osteogenic, antioxidant, anti-inflammatory

## Abstract

Polyphenols are natural bioactives occurring in medicinal and aromatic plants and food and beverages of plant origin. Compared with conventional therapies, plant-derived phytochemicals are more affordable and accessible and have no toxic side effects. Thus, pharmaceutical research is increasingly inclined to discover and study new and innovative natural molecules for the treatment of several chronic human diseases, like type 2 diabetes mellitus (T2DM) and osteoporosis. These pathological conditions are characterized by a chronic inflammatory state and persistent oxidative stress, which are interconnected and lead to the development and worsening of these two health disorders. Oral nano delivery strategies have been used to improve the bioavailability of polyphenols and to allow these natural molecules to exert their antioxidant, anti-inflammatory, anti-diabetic, and pro-osteogenic biological activities in in vivo experimental models and in patients. Polyphenols are commonly used in the formulations of nutraceuticals, which can counteract the detrimental effects of T2DM and osteoporosis pathologies. This review describes the polyphenols that can exert protective effects against T2DM and osteoporosis through the modulation of specific molecular markers and pathways. These bioactives could be used as adjuvants, in combination with synthetic drugs, in the future to develop innovative therapeutic strategies for the treatment of T2DM and osteoporosis.

## 1. Introduction

**Figure 1:**
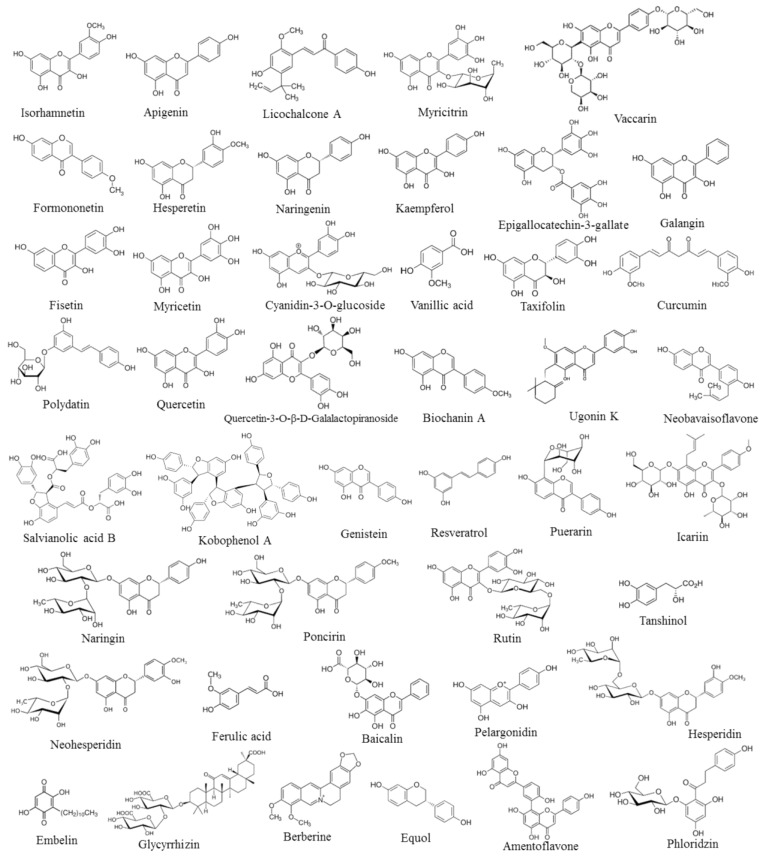
Chemical formulae of the polyphenols reported in this study.

Phytochemicals are natural molecules found in plants and foods, especially fruits and vegetables. Polyphenols (like quercetin, naringenin, hesperetin, and polydatin) are listed among phytochemicals along with phenylpropanoids, terpenoids, alkaloids, and quinones [1]. Polyphenols possess several biological properties: antioxidant, anti-inflammatory, chemopreventive, anti-insulin resistance, and pro-osteogenic [2,3,4,5] and are able to exert pleiotropic activities, meaning that they can target multiple molecular markers of different molecular pathways at the same time [6]. Flavonoids are listed among polyphenols along with phenolic acids, stilbenes, and lignans, as well as other less common types such as tannins, coumarins, quinones, flavonolignans, and curcuminoids. Flavonoids are the most abundant polyphenols found in the human diet, and this class of compounds is further grouped into flavones, flavonols, flavan-3ols, isoflavones, anthocyanidins, and flavanones based on their different chemical structures [7]. It should be noted that the structure-activity relationship (SAR) mechanisms are important to understanding the biological activities of phytochemicals and, in particular, of polyphenols. Notably, the Transient Receptor Potential Canonical (TRPC) channels are mechanisms for enabling the entry of Ca^2+^ and Na^+^ into cells and are involved in the induction of cell proliferation [8]. Interestingly, upon adipocyte maturation, there is an upregulation of constitutively active TRPC1 and TRPC5 heteromeric channels [8]. Since an increase in adipocyte maturation and differentiation is associated with osteoporosis development [5], natural molecules that are able to inhibit TRPC channels can exert anti-osteoporotic effects. Notably, the polyphenol resveratrol is an indirect inhibitor of TRPC5, while the stilbene diethylstilbestrol is a direct inhibitor of TRPC5. One of the main differences between these two phytochemicals is the presence of two ethyl groups on diethylstilbestrol, which causes a difference in the preferred conformation of the aromatic rings of this polyphenol when compared with resveratrol, influencing the biological activities of diethylstilbestrol [8]. Interestingly, Ninfali et al. [2] described the anti-inflammatory, antioxidant, and antitumor properties of several polyphenols, belonging to the class of C-glycosyl flavonoids, and attributed these activities to their chemical structure, which allows these bioactives to interact with enzymes that are involved in the mechanisms of oxidative stress, chronic inflammatory state, and cancer development [2]. All flavonoids have 15 carbon atoms in their fundamental nucleus C6-C3-C6 structure, with several substituted chemical groups [9]. Since flavonoids comprise the same core scaffold, the functional variation concerning their anti-inflammatory, antioxidant, and anti-diabetic activities is largely associated with the presence of different substituent groups in the different positions of the flavonoid’s skeleton [9]. Interestingly, researchers have evaluated the inhibitory activity of several flavonoids in terms of the transcription of the pro-inflammatory cyclooxygenase-2 (*COX-2*) gene, and the experimental results indicated that the presence of a 4-oxo group in the C-ring and a 3′-4′-dihydroxy structure in the B-ring is required for exerting the anti-inflammatory activity [10]. Notably, it was indicated that flavonoids can exert their anti-inflammatory activity through the inhibition of the lipoxygenase (LOX) enzyme [11]. It was reported that the flavonoids’ LOX inhibitory effect is attributable to the C2-C3 double bond in the C-ring and the hydroxyl groups at C-3′ and C-4′ in the B-ring [11]. Particularly, it was shown that the chemical structure of flavonoids also plays a pivotal role in the inhibition of the activity of the pro-inflammatory markers NF-kB and inducible nitric oxide synthase (iNOS) [12]. The authors reported that the C2-C3 double bond can influence the anti-inflammatory activity of these flavonoids. In addition, the -OH groups at C3′ and C4′ and the presence of a sugar moiety in the A-ring lead to better anti-inflammatory activity. Interestingly, it was shown that the antioxidant activity and reduction of the ROS levels of several flavonoids extracted from mulberry leaves were associated with the -OH groups of these bioactives [9]. Notably, flavonoids have been reported to possess anti-diabetic effects through various mechanisms of action [9]. Interestingly, it was shown that the ability of flavonoids to decrease the levels of glycation end-products (AGEs) was determined by the presence of hydroxyl groups at C3′ and C4′ of the B-ring, as well as the C5 and C7 locations of the A-ring [13]. Notably, researchers have evaluated the inhibition of α-glucosidase activity exerted by flavonoids and reported that the presence of hydroxyl groups at the C5 and C7 or C8 positions of the A-ring is important for this biological activity. Moreover, the hydroxyl groups at C3′ and C4′ of the B-ring, the C2-C3 double bond, and the hydroxyl group at C3 were also fundamental for the inhibition of the α-glucosidase catalytic activity [14]. Interestingly, researchers isolated several flavonoids from *Tetracera indicia* and *Tetracera scandens* extracts and evaluated their ability to inhibit the catalytic activity of the DPPIV enzyme, which plays a pivotal role in the development of insulin resistance mechanisms [15]. The experimental results indicated that the presence of hydroxyl groups, the C2-C3 double bound, and the 4-oxo group were important for the inhibition of DPPIV activity [15]. Furthermore, it was shown that flavonoids benefit from the presence of the C2-C3 double bond and the hydroxyl groups of the C3′, C4′, C5, and C7 positions for exerting both anti-inflammatory and anti-diabetic effects concurrently [9]. Figure 1 shows the chemical formulae of the polyphenols described in this study, which have been selected based on their well-documented anti-diabetic and anti-osteoporotic activities reported in the literature and on the demonstrated possibility of increasing their bioavailability and biological properties through innovative nano delivery technologies.

Several polyphenols are commonly used in the formulations of nutraceuticals because of their complex biological potential, exerted through the ability to interact with enzymes, transcription factors, and signaling molecules involved in the development of several chronic human diseases [2,16,17]. Nutraceuticals have been recognized to operate within a distinct clinical space that is ‘before drugs, beyond the foods’. This delineates their role as agents that bridge the gap between basic nutritional foodstuffs and pharmaceuticals. Even if they are not classified as drugs, due to their derived nature from plant sources and their less intensive regulatory requirements, nutraceuticals still hold potential therapeutic efficacy that can be employed in preventive healthcare or as an adjunct in disease management, often before the initiation of conventional drug therapy [18]. GliceFen^®^ (Mivell, Fano, Italy) and BlastiMin Complex^®^ (Mivell, Fano, Italy) are examples of nutraceuticals characterized by a formulation based on polyphenols. Naringenin and hesperetin of the GliceFen^®^ formulation target the insulin resistance mechanisms of T2DM [4], while the combination of curcumin, polydatin, and quercetin, which is present in both the GliceFen^®^ and BlastiMin Complex^®^ nutraceuticals, possesses antioxidant and anti-inflammatory properties, exerted through the reduction of ROS, IL-1β and IL-8 levels [3]. Furthermore, the combination of curcumin, polydatin, and quercetin with orthosilicic acid and vitamin K2 present in the nutraceutical BlastiMin Complex^®^ can induce pro-osteogenic effects in in vitro experimental models and decrease the levels of the pro-inflammatory factors p-P38 and p-NF-kB, which play a role in the development of many chronic diseases [2,5]. The protection associated with nutraceutical use and polyphenol consumption is determined mainly by their antioxidant and anti-inflammatory biological effects. Under physiological conditions, pro-oxidant molecules are neutralized by enzymatic and non-enzymatic antioxidants. Enzymatic antioxidants superoxide dismutase (SOD), catalase (CAT), and glutathione peroxidase (GPx) counteract the damage exerted by radical oxygen species (ROS) on proteins, lipids, and DNA [19]. ROS are formed as natural by-products of cellular metabolism and are involved in the induction of oxidative stress and the subsequent initiation of cell death. Oxidative stress is caused by a perturbation of pro-oxidants (like ROS) and the antioxidant mechanisms, leading to the excessive production of pro-oxidants relative to antioxidant defenses [20,21]. Oxidative phosphorylation, plasma membrane proteins such as nicotinamide adenine dinucleotide phosphate oxidase (NOXs), peroxisomes, and the enzyme cyclooxygenases contribute to the increase in pro-oxidant levels in the cells [22]. Notably, ROS can induce the activation of the pro-inflammatory transcription factor Nuclear Factor-kappa B (NF-kB), indicating that the pro-inflammatory and pro-oxidant molecular pathways are interconnected [23,24]. NF-kB is also able to induce the expression of genes involved in oxidative stress, like NADPH oxidase *NOX2* [25,26]. Interestingly, it has been demonstrated that oxidative stress and inflammation interact with hyperglycemia, leading to the development of both T2DM and pathologies associated with diabetes [22,27]. Notably, the pro-inflammatory markers C-reactive protein (CRP), interleukin-1β (IL-1β), interleukin-6 (IL-6), NF-kB, and tumor necrosis factor-alpha (TNF-α) contribute to the chronic inflammatory state of T2DM and are significantly elevated in patients with diabetes [28,29,30,31].

**Figure 2:**
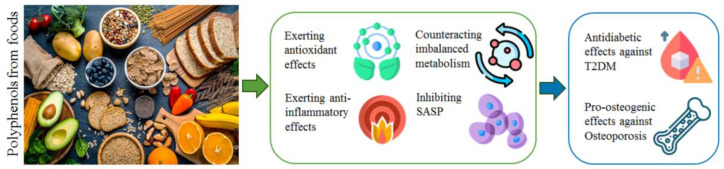
Biological activities and molecular mechanisms modulated by polyphenols obtained from foods and several plant sources and that lead to the inhibition of T2DM and osteoporosis development. T2DM: Type 2 diabetes mellitus; SASP: senescence-associated secretory phenotype. The icons used for Figure 2 are free and have been downloaded from https://www.flaticon.com/ (accessed on 20 June 2024) [75].

Interestingly, it has been reported that the chronic inflammatory state can also contribute to osteoporosis development. In particular, increased levels of inflammatory cytokines, such as TNF-α, IL-1, IL-6, interleukin-7 (IL-7), and interleukin-17 (IL-17) stimulate the expression of the receptor activator of the nuclear factor-kappa B ligand (RANKL), favoring osteoclastogenesis [32]. In addition, it has been shown that increased levels of ROS favor bone tissue degradation by inhibiting osteoblastogenesis and inducing osteoclastic differentiation, indicating that both oxidative stress and pro-inflammatory mechanisms contribute to osteoporosis development [33,34]. Nutraceutical formulations are composed of natural molecules that possess remarkable antioxidant and anti-inflammatory properties. Nutraceutical treatments can be used as adjuvants in combination with synthetic drugs to complement pharmacological therapies and inhibit the development of chronic diseases in subjects that might not qualify for conventional pharmacological treatments but could benefit from therapeutic approaches based on natural molecules [35]. Recently, Gonzalez et al. [36] and Heidarzadeh-Esfahani et al. [37] described the anti-diabetic effects of polyphenols in their works. These authors reported on the biological activities of polyphenols concerning the protection of pancreatic islet β-cells, the antioxidant capacities of these bioactives, the effects on insulin secretion, the regulation of intestinal microbiota [36], and the effects of a diet rich in polyphenols in a cohort of 7000 participants, indicating that a high plant-based diet index (PDI) can be recommended to reduce the risk of T2DM development [37]. Notably, Zeng [38] and Wen et al. [39] reported the pro-osteogenic effects of polyphenols. These authors described the antioxidant, anti-inflammatory, and anti-bone resorption effects of polyphenols [38] and also the polyphenol-mediated modulation of bone metabolism and metabolites produced by intestinal flora using in vivo experimental models [39]. However, none of these authors described the remarkable connection between the molecular pathways that lead to T2DM and osteoporosis pathological conditions and how the polyphenols modulate the molecular markers that play a role in the development of both of these two chronic human diseases. Furthermore, in these works, the authors did not report on the nano delivery strategies used to improve the bioavailability and biological activities of polyphenols, an important parameter by which to obtain remarkable results in experimental models and clinical trials. Our review describes the polyphenols extracted from several plant sources (Table 1), which can exert antioxidant, anti-inflammatory, anti-diabetic, and pro-osteogenic effects against T2DM and osteoporosis (Figure 2) through the modulation of specific and interconnected molecular markers and pathways in both in vitro and in vivo experimental models, the nano delivery technologies utilized to improve the bioavailability of these bioactives, and the clinical trials that have reported the results obtained after the administration of polyphenols in diabetic and osteoporotic patients.

**Table 1:** Plant sources of the polyphenols reported in this review.

Polyphenols	Plant Sources	References
Isorhamnetin	*Tetrastigma hemsleyanum*	[40]
Apigenin	*Eclipta alba*	[41]
Licochalcone A	*Glycyrrhiza glabra*	[42]
Myricitrin	*Myrica esculenta*	[43]
Biochanin A	*Trifolium pratense*	[44]
Formononetin	*Trifolium pratense*	[45]
Hesperetin	*Citrus wilsonii*	[46]
Naringenin	*Citrus grandis*	[47]
Kaempferol	*Moringa oleifera Lam*	[48]
Epigallocatechin-3-gallate	*Camellia sinensis*	[49]
Galangin	*Rhizoma alpiniae*	[50]
Fisetin	*Carica papaya*	[51]
Myricetin	*Marcetia taxifolia*	[52]
Cyanidin-3-O-glucoside	*Sambucus nigra*	[53]
Vaccarin	*Vaccariae semen*	[54]
Taxifolin	*Pseudotsuga menziesii*	[55]
Curcumin	*Curcuma longa*	[47]
Polydatin	*Polygonum multiflorum*	[56]
Quercetin	*Embelia Ribes*	[57]
Quercetin-3-O-β-D-galactopiranoside	*Byrsonima crassa Niedenzu*	[58]
Vanillic acid	*Sambucus williamsi Hance*	[59]
Ugonin K	*Helminthostachys zeylanica*	[60]
Neobavaisoflavone	*Psoralea corylifolia*	[61]
Salvianolic acid B	*Salvia miltiorrhiza*	[62]
Kobophenol A	*Coragana sinica*	[63]
Genistein	*Genista tinctoria*	[47]
Resveratrol	*Polygonum cuspidatum*	[4]
Puerarin	*Pueraria lobata*	[64]
Icariin	*Herba epimedium*	[65]
Tanshinol	*Salvia miltiorrhiza*	[66]
Naringin	*Citrus wilsonii*	[46]
Poncirin	*Poncirus trifoliata*	[67]
Rutin	*Tetrastigma hemsleyanum*	[40]
Neohesperidin	*Citrus wilsonii*	[46]
Hesperidin	*Citrus wilsonii*	[46]
Baicalin	*Scutellaria baicalensis*	[68]
Ferulic acid	*Ferula foetida*	[69]
Glycyrrhizin	*Glycyrrhiza glabra*	[42]
Berberine	*Mahonia Leschenaultia*	[47]
Pelargonidin	*Glycine max Merr*	[70]
Embelin	*Embelia ribes*	[71]
Amentoflavone	*Selaginella tamariscina*	[72]
Phloridzin	*Malus domestica*	[73]
Equol	*Trifolium pratense*	[74]

## 2. Insights into Molecular Targets Modulated by Polyphenols

### 2.1. Molecular Pathways Modulated by Polyphenols in Type 2 Diabetes Mellitus

Diabetes mellitus is a chronic metabolic disorder that represents a major global health issue [76,77]. Type 1 diabetes mellitus affects 5% of diabetic patients, while the remaining 95% are affected by T2DM [78]. This pathology is associated with several clinical complications [79] and is characterized by an insulin deficit caused by the pancreatic dysfunction of β-type cells, the development of insulin resistance in target organs, decreased protein levels of insulin receptors, and a chronic inflammatory state [80]. Notably, the hormone insulin regulates blood glucose levels and promotes glucose uptake via glucose transporter 4 (GLUT4) translocation in skeletal muscles and adipose tissue, leading to an increase in hepatic glycogen levels. Particularly, it has been shown that AGEs can contribute to the induction of oxidative stress, leading to a decrease in insulin secretion and GLUT4 translocation, an increase in blood glucose levels, and hyperglycemia development [22].

Notably, it has been reported that insulin signaling starts with the binding of this hormone to the insulin receptor (INSR) present on the cell surface. This interaction leads to the tyrosine phosphorylation of insulin receptor substrate (IRS) proteins, which assemble a signaling complex that includes several proteins, such as phosphoinositide 3-kinase (PI3K) [4]. This event determines an increase in phosphatidylinositol 3,4,5-trisphosphate levels and, consequently, the recruitment of the kinases AKT and 3-phosphoinositide-dependent kinase 1 (PDK1), which phosphorylates AKT, leading to its activation. AKT plays a pivotal role in the insulin signaling pathway and mediates most of the physiological metabolic actions of insulin. Notably, AKT can induce GLUT4 translocation by activating the TBC1 domain family member 4 (TBC1D4)/RAB10 molecular pathway, which induces the translocation of GLUT4 to the cell membrane, leading to an increase in glucose uptake by the cells and a decrease in blood glucose levels [4]. Considering this scientific evidence, the polyphenols that can increase GLUT4 and INSR levels, activate the IRS/PI3K/Akt molecular pathway, and inhibit the molecular mechanisms of oxidative stress through the activation of antioxidant cellular defenses can inhibit T2DM development and the onset of complications related to diabetes, such as blood vessel destruction, damage to the heart, eyes, kidneys, and central nervous system [81,82]. If the molecular mechanisms that lead to the development of diabetes complications are not inhibited, both macrovascular (atherosclerotic) and microvascular (retinopathy, nephropathy, and neuropathy) disorders occur. Notably, hyperglycemia casts an additional inflammatory burden on the pancreatic islets by stimulating the production of pro-inflammatory cytokines that are regulated by the transcription factor NF-kB (like TNF-α [83]) and lead to the development of the senescence-associated secretory phenotype (SASP) [84]. Notably, the SASP also contributes to diabetes pathology and the onset of diabetes complications [84,85,86].

The main objective of T2DM management by clinicians is to achieve optimal glycaemic control and prevent or delay the development and worsening of diabetic complications. Dietary and lifestyle modifications are recommended approaches for managing T2DM, together with pharmacological interventions including oral hypoglycemic medications (e.g., glipizide, metformin, or acarbose) or, ultimately, insulin injections [87]. Notably, emerging preclinical and clinical evidence has demonstrated the remarkable benefits of therapies using natural products in treating hyperglycemia, β-cell dysfunction, and insulin resistance in diabetes [86,88]. In fact, polyphenols contained in many foods can exert both anti-inflammatory and antioxidant biological effects, thus potentially protecting against T2DM development and T2DM-related complications [89,90]. A recent study by Farzaei et al. further suggested that flavonoids are especially effective in targeting inflammation, a key factor in metabolic disorders like T2DM. These compounds mitigate inflammation-related pathways, thereby offering a promising integrative strategy for managing and preventing metabolic disorders [91].

### 2.2. Polyphenols for T2DM Treatment

Notably, several studies have reported an increase in the use of natural products for the treatment of patients with T2DM [91,92,93,94]; this is due to the long-term use of insulin and oral hypoglycemic drugs, which are characterized by numerous side effects that include nausea, vomiting, diarrhea, and hepatorenal disorders [95]. Compared with conventional therapies, natural plant-derived agents are more affordable and accessible, without toxic side effects, so pharmaceutical research is increasingly interested in studying innovative anti-diabetic natural molecules [96].

To this end, the important role that isorhamnetin plays in lowering glucose concentrations by increasing GLUT4 levels, improving oxidative status through the increase in Nrf2, SOD, and CAT levels, reducing inflammation by decreasing NF-kB, and adjusting lipid metabolism in both in vitro and in vivo models suggests that isorhamnetin may be used for the treatment of T2DM [97]. Interestingly, apigenin exerts strong antioxidant, anti-diabetic, and anti-inflammatory actions, which are mediated through neutralizing ROS, increasing SOD and CAT activity, reducing NF-kB levels, and inhibiting the α-glucosidase enzyme [22,98]. Notably, the flavonoid licochalcone A can counteract the detrimental effects of diabetic nephropathy both in in vitro and in vivo experimental models through the activation of the IRS-2/PI3K/Akt pathway [42]. Notably, the flavone myricitrin can remarkably decrease blood glucose levels in T2DM in vivo models [43]. This phytochemical increases glucose absorption by skeletal muscles through the activation of Insulin Receptor Substrate-1 (IRS-1)/Phosphoinositide 3-kinase (PI3K)/Ak strain-transforming (Akt)/GLUT4 signaling, as evidenced by both in vitro and in vivo studies. Moreover, myricitrin inhibits NF-kB, decreases ROS levels, and increases the activity of the antioxidant transcription factor nuclear factor erythroid 2-related factor 2 (Nrf2) in both in vitro and in vivo studies [99,100]. Biochanin A is an isoflavone that possesses anti-inflammatory, anti-hyperlipidemic, and antioxidant properties. In animal models of T2DM, this phytochemical causes increased insulin sensitivity, decreases glycohemoglobin A1C (HbA1c) formation, and inhibits the pro-inflammatory marker NF-kB [101]. It has been shown that formononetin treatment reduces hyperglycemia in pancreatic cells by increasing the levels of Sirtuin 1 (SIRT1), an enzyme that protects cells from ROS-induced damage [102]. Hesperidin is a flavonoid compound found in citrus fruits, and it can exert anti-diabetic effects by regulating SIRT1, alleviating inflammation, counteracting oxidative stress through an increase in SOD and GPx levels, and reducing insulin resistance [103]. Naringenin, a flavonoid compound found in several citrus fruits, exhibits potent anti-hyperglycemic and anti-hyperlipidemic properties in diabetic rat models [104]. The actions of naringenin include improving hyperglycemia, insulin sensitivity, pancreatic cell performance, and lipid profile [104]. Furthermore, naringenin possesses both antioxidant properties exerted through the increase in SOD, CAT, and SIRT1 levels and anti-inflammatory properties, associated with a reduction in TNF-α levels [104]. Interestingly, the anti-diabetic effects of kaempferol are exerted by targeting multiple pathways, including improving glucose uptake, glycogen synthesis, adenosine monophosphate-activated protein kinase (AMPK) activity, and GLUT4 expression. Additionally, several in vivo studies have reported the anti-diabetic effects of kaempferol, which decreased plasma glucose levels and increased plasma insulin levels [105], decreased glucose synthesis [106], increased glucagon-like peptide 1 (GLP-1) levels, and enhanced insulin release [107,108]. This phytochemical showed potent anti-inflammatory effects by reducing nucleic and cytosolic levels of NF-kB, TNF-α, and IL-6, and it also regulated the phosphorylation of IRS-1 [108]. Notably, epigallocatechin-3-gallate (EGCG) exerted antihyperglycemic, antidyslipidemic, anti-inflammatory, and antioxidant actions in an in vivo model through the increase in SOD and CAT activity and the decrease in HbA1c and IL-1β levels [109]. The flavonoid galangin is a potent inhibitor of dipeptidyl peptidase IV (DPPIV), an integral membrane enzyme expressed in cells [110], which cleaves the incretin hormone GLP-1, which is responsible for the maintenance of normal glucose homeostasis [111]. Notably, fisetin can inhibit the gluconeogenesis process and glycogen breakdown, leading to a decrease in blood glucose and HbA1c levels [112,113]. The flavonoid myricetin is widely distributed in different types of fruits and herbs, and this phytochemical can inhibit the catalytic activity of DPPIV and is a GLP-1 receptor agonist [114]. In addition, myricetin can increase the levels of the antioxidant enzymes CAT and SOD [114]. Interestingly, anthocyanins, like cyanidin-3-O-glucoside, regulate digestive enzymes (α-amylase and α-glucosidase), GLUT-4, and GLP-1 levels. Furthermore, these bioactives normalize insulin secretion and counteract insulin resistance conditions [115]. The additional anti-diabetic mechanisms of anthocyanins involve the protection of pancreatic β cells through their anti-inflammatory (decrease in TNF-α levels) and antioxidant (increase in SOD activity) properties [116]. Vaccarin, a potent flavonoid glycoside extracted from *Vaccariae Semen*, is able to decrease blood glucose levels, reduce oxidative stress by increasing GPx levels, and inhibit the molecular mechanisms of insulin resistance in T2DM in vivo models [54,117]. Notably, the flavonoid taxifolin can inhibit the α-amylase enzyme and counteract hyperglycemia conditions via the inhibition of oxidative stress, exerted through the induction of SOD, CAT, and GPx activity [118,119]. Notably, it has been reported that phloridzin (a polyphenol isolated from apples) exerts antidiabetic effects in vitro through the induction of AMPK and Akt phosphorylation and the inhibition of insulin resistance molecular mechanisms [120].

Interestingly, it has been shown that the combination of curcumin, polydatin, quercetin, naringenin, and hesperetin present in the nutraceutical GliceFen^®^ (Mivell, Fano, Italy) was able to synergistically and significantly decrease the expression levels of the pro-inflammatory gene Semaphorin 3E (*SEMA3E*), which is also involved in the mechanisms of insulin resistance through the inhibition of Akt phosphorylation [4,121]. This formulation was also able to synergistically and significantly inhibit the catalytic activity of the hyperglycemic enzyme DPPIV [4], which plays a pivotal role in the development of a hyperglycemia condition in T2DM [122]. Notably, orally active DPPIV inhibitors are increasingly used in new therapies for T2DM, and these drugs lead to an increase in the levels of the hormone GLP-1, which is involved in the stimulation of insulin secretion and the increase in insulin sensitivity in tissues [123,124,125]. Particularly, the ability of the phytochemicals of the nutraceutical GliceFen^®^ (Mivell, Fano, Italy) to reduce Semaphorin 3E protein levels, increase insulin receptor (INSR) levels, and inhibit DPPIV catalytic activity identifies this nutraceutical as a natural adjuvant for innovative T2DM therapeutic approaches in combination with synthetic drugs [4].

### 2.3. Molecular Mechanisms Modulated by Polyphenols in Osteoporosis

Bone is a tissue composed of a mineralized organic matrix and different types of cells [126,127]. In the bone tissue environment, specialized cells, such as osteoblasts and osteoclasts, are responsible for bone matrix secretion and resorption, respectively [128]. Proteins synthesized by osteoblasts, such as alkaline phosphatase (ALP) and osteocalcin (OCN), contribute to matrix mineralization [129,130]. Moreover, the transcription factors runt-related transcription factor 2 (Runx2) and Osterix (OSX) play a pivotal role in inducing the process of osteoblastic differentiation in bone marrow mesenchymal stem cells (BM-MSCs), which are involved in maintaining bone health [131,132]. Notably, with aging, there are several pathological modifications in the microenvironment of bone tissue, and among these modifications is the accumulation of senescent cells. Senescent BM-MSCs acquire distinctive phenotypic and metabolic alterations, named SASP, characterized by pro-inflammatory activity, which has been hypothesized to be the leading cause of tissue dysfunction and bone loss in osteoporosis [5]. Interestingly, it has been demonstrated that senescent BM-MSCs can cause paracrine senescence in young BM-MSCs via the production of inflammatory cytokines and the activation of the NF-kB pathway [5]. Considering this scientific evidence, the polyphenols that can increase Runx2, ALP, OCN, and OSX levels and counteract the ability of senescent BM-MSCs to release those cytokines and interleukins that can transform the young BM-MSCs into senescent BM-MSCs, play a pivotal role in counteracting osteoporosis development and in avoiding the depletion of the cell population of young BM-MSCs, which can differentiate into new osteoblasts and induce the formation of new bone tissue [5]. Notably, bone morphogenetic proteins (BMPs) and Wingless-type MMTV integration site family (Wnt)/β-catenin signaling are the main molecular pathways responsible for the modulation of transcription factors related to osteoblastic differentiation and the generation of new bone tissue [131,133]. On the contrary, osteoclasts promote tissue resorption through the secretion of acids and proteolytic enzymes into the bone matrix, like the cysteine proteases cathepsin K and matrix metalloproteinases (MMPs) [134,135,136]. Osteoclastogenesis is regulated by RANKL, the receptor activator of nuclear factor-kB (RANK), osteoprotegerin (OPG), and monocyte colony-stimulating factor (M-CSF) [134]. Osteoblasts produce RANKL to recruit osteoclasts and induce the bone remodeling process. In fact, RANKL/RANK interaction increases osteoclast differentiation, activity, and survival [137]. On the contrary, OPG interacts with RANKL and acts as a decoy receptor, reducing the proliferation of osteoclasts [134]. Interestingly, after the binding of the ligand RANKL with its receptor RANK, the release of the pro-inflammatory cytokines TNF-α, IL-1, and IL-7 is stimulated [138,139] and the recruitment of the transcription factors Fos proto-oncogene (cFos) and the nuclear factor of activated T-cells cytoplasmic 1 (NFATc1) is activated, leading to osteoclast differentiation [140,141]. Notably, the RANKL/OPG ratio and crosstalk between osteoblasts and osteoclasts directly influence bone turnover and remodeling processes [32,137,142]. Bone loss happens when the rate of resorption exceeds the rate of new bone formation, leading to osteoporosis development [143,144]. Osteoporosis has become a global health issue, and it is defined as a condition characterized by micro-architectural deterioration and the low mineral density of bone tissue, resulting in enhanced bone fragility and an increased risk of fracture [142]. Interestingly, it has been shown that osteoporosis incidence and progression are significantly influenced by oxidative stress [145,146]. Notably, postmenopausal osteoporosis is an age-related systematic metabolic disorder that affects an increasing number of patients [147]. Bone diseases like osteoporosis necessitate the artificial upregulation of bone mineral density (BMD) with the aid of therapeutic strategies based on drugs and/or natural products. Conventional therapies utilized for the treatment of bone pathologies are characterized by adverse effects like a burning sensation and gastrointestinal tract disturbances, limiting the use of these therapies [148]. The drugs that are used for the treatment of osteoporosis include anti-bone resorption therapies, represented by bisphosphonates (alendronate, clodronate) [149], cathepsin K inhibitors, and selective estrogen receptor modulators (SERMs). Moreover, vitamin D3, the parathyroid hormone analog Teriparatide, and RANKL inhibitors (the antibody Denosumab) are among the medications that counteract osteoporosis pathological mechanisms [150]. These medications possess side effects such as hypercalcemia, endometrial and breast cancer development, hot flushes, hypercalciuria, painful breasts, and thromboembolic events [151]. Notably, it has been shown that phytochemicals and plant extracts can play a role in the improvement of bone health and do not exert the toxic side effects associated with therapies based on synthetic drugs [152,153]. Interestingly, it was reported that polyphenols present in several foods can increase BMD values, inhibit bone resorption mechanisms, and enhance the activity of osteoblasts [32,33].

### 2.4. Polyphenols for Osteoporosis Treatment

In bone tissue, the inhibitory effects of polyphenols on oxidative stress and on the inflammatory process increase the survival of osteoblasts and modulate osteoclastic differentiation [46,154]. Polyphenols exert a regulatory effect on osteoclastic activity by inhibiting the expression of markers involved in bone resorption, such as RANKL and proteolytic enzymes, including MMPs, cathepsin K, and tartrate-resistant acid phosphatase (TRAP) [154]. Additionally, polyphenols can stimulate the expression of osteogenic markers related to osteoblast differentiation and bone matrix mineralization, such as Runx2, ALP, OCN, type 1 collagen (COL-1), osteopontin (OPN), and BMP-2 [32,33,46,154]. Interestingly, it has been shown that the natural molecule fisetin was able to increase the levels of the osteogenic markers collagen type 1 alpha 1 (COL1α1), OCN, OPG, and RUNX2 in pre-osteoblastic MC3T3-E1 cells [155]. Notably, in human BM-MScs, quercetin-3-O-β-D-galactopyranoside increased ALP catalytic activity and the levels of RUNX2 and OCN, leading to the stimulation of the osteoblastogenesis process [156]. Vanillic acid is a phenolic acid, abundantly found in *Sambucus williamsii* Hance. This phytochemical, at a dose of 100 mg/kg, increased ALP, OCN, and BMD levels in ovariectomized (OVX) rats [59]. Moreover, vanillic acid exerted anti-inflammatory effects by reducing IL-1β, IL-6, and TNF-α levels [59]. Ugonin K is a flavonoid extracted from the roots of *Helminthostachys zeylanica* (L.) Hook. An in vitro study in MC3T3-E1 osteoblastic cells demonstrated that ugonin K remarkably increased ALP activity and enhanced OCN, OSX, and RUNX2 protein levels [60]. This polyphenol was also able to inhibit ROS generation and decrease caspase activity levels in osteoblasts [157]. Neobavaisoflavone is a typical isoflavone isolated from *Psoralea corylifolia* L. This phytochemical promoted osteogenesis in MC3T3-E1 cells through the increase in COL1α1, OCN, OSX, and RUNX2 levels and an enhancement of ALP activity [61]. Salvianolic acid B is a major phenolic derivative found in *Salvia miltiorrhiza* Bunge. In an in vitro model of human mesenchymal stem cells, salvianolic acid B stimulated the mineralization process and upregulated ALP, RUNX2, OPN, and OSX [62]. When tested in an in vivo model at a dose of 80 mg/kg/day, this polyphenol inhibited the osteopenia condition and increased bone mass, bone thickness, ALP activity, and collagen I protein levels [158]. Kobophenol A is a tetrameric stilbene obtained from the plant *Caragana sinica* (Buc’hoz) Rehder. This bioactive stimulated the proliferation of human osteoblasts, increased ALP activity, and counteracted oxidative stress [63]. Formononetin is an isoflavone extracted from *Butea monosperma* (Lam.) Kuntze. In in vivo experimental models, this polyphenol induced an increase in Runx2 and OCN protein levels and upregulated the expression levels of the genes *ALP*, *OCN*, *OPN*, and *COL1A1*, indicating osteogenic differentiation [159,160]. Genistein is an isoflavone extracted from *Genista tinctoria* L. Interestingly, the combination of genistein and zinc exerted pro-osteogenic effects in OVX rats and an increase in lumbar spine and femur BMD [161]. In addition, genistein upregulated Wnt/β-catenin, Runx2, PPARγ, and BMP2 markers and downregulated the pro-inflammatory factors transforming growth factor-beta (TGF-β) and NF-kB, leading to osteoblast differentiation [162,163]. Resveratrol is a polyphenolic compound widely recognized for its presence in peanuts, grapes, red wine, and some berries, demonstrating its diverse natural sources beyond just red grapes. This phytochemical increased BMD and the biomechanical properties of the bone tissue in an in vivo model [164]. Moreover, resveratrol was able to reduce the levels of the bone resorption markers RANKL and TRAP-5b and increase OPG levels [164]. Furthermore, an in vivo study showed that resveratrol significantly inhibited bone loss and augmented the levels of osteogenic markers Col1α1, Runx2, and OCN [142]. Polydatin is the glycosylated form of resveratrol. Interestingly, it has been demonstrated that, in an in vitro model of MC3T3-E1 cells, polydatin treatment significantly increased the expression levels of the genes *COL1A1*, *ALP*, *OCN*, *RUNX2*, and *ALP* [56]. In addition, polydatin treatment decreased the protein levels of pro-inflammatory markers p-p38 and phospho-Jun N-terminal kinase (p-JNK), and reduced the protein levels of phospho-extracellular signal-regulated kinase (p-ERK), which is involved in osteoclast activation [56]. Puerarin is a phytoestrogen found in *Pueraria lobata* (Willd.) Ohwi. Notably, it has been reported that the combination of puerarin and zinc in OVX rats increased OPG, OPN, and calcium levels and decreased RANKL levels [165]. Icariin is a flavonoid glycoside obtained from *Herba epimedium*. This polyphenol inhibited the differentiation and activity of osteoclasts and induced the proliferation of osteoblasts by activating the molecular pathway cAMP/PKA/CREB [65]. Tanshinol is a polyphenol abundantly found in the roots of *S. miltiorrhiza*. This bioactive was able to regulate the Wnt/β-catenin/RUNX2 molecular pathway and improve the biomechanical properties of bone tissue in vivo [66]. Naringin is a polymethoxylated flavonoid isolated from grapefruit. This phytochemical enhanced BMP-2 expression and induced osteoblast differentiation through the modulation of Akt, PI3K, c-Fos/c-Jun, and Activator Protein-1 (AP-1) markers. Furthermore, naringin treatment increased ALP activity, OPN synthesis, and OCN protein levels [166]. Moreover, in in vitro experiments, naringin decreased both RANKL and NF-kB levels, leading to an induction of the apoptotic process in osteoclasts [167], while in an in vivo model this bioactive improved BMD, bone mineralization, and bone mechanical strength [167]. Poncirin is a flavonoid obtained from the fruit *Poncirus trifoliata* (L.) Raf. In an in vivo experimental model, this polyphenol increased OPG and OCN levels, BMD values, and decreased C-terminal telopeptide (CTX) levels [67]. Kaempferol is a flavonoid derived from the rhizome of *Kaempferia galanga* L.; this phytochemical was able to increase the levels of BMP-2, RUNX2, OSX, and collagen in osteoblasts [168]. Notably, kaempferol inhibited osteoclast formation through a decrease in p38, ERK1/2, and JNK MAP kinase phosphorylation and a reduction in NFATc1 levels [169]. Rutin is a flavonoid found in citrus fruits. An in vivo study showed that the administration of rutin significantly improved BMD, increased the levels of ALP and OCN, and decreased the levels of inflammatory markers like IL-6, TNF-α, and Interferon-gamma (IFN-γ) [170]. Notably, it was shown that neohesperidin inhibited osteoclast differentiation through a decrease in NF-kB activity and a reduction in RANKL, cathepsin K, and TRAP levels in vivo [171]. Particularly, it was also reported that this polyphenol remarkably increased MSC proliferation, ALP activity, and the expression of the osteogenic markers Runx2, OCN, BMP-2, and β-catenin. Interestingly, the effects of neohesperidin were blocked by the addition of Dickkopf (DKK1), an antagonist of the Wnt/β-catenin pathway, which was also inhibited by sclerostin [172,173]. It was reported that hesperidin exerted anti-inflammatory effects through a decrease in IL-1β, IL-6, and TNF-α levels, significantly increased BMD, and inhibited bone loss by increasing RUNX2, ALP, and OCN levels in in vivo experimental models [173,174]. Notably, it has been shown that hesperetin increased the proliferation and activity of osteoblasts in vitro and induced the expression of the osteogenic markers BMP-2, Runx2, and OSX [175]. Furthermore, in human BM-MSCs, hesperetin was able to upregulate the protein levels of ALP, Runx2, OCN, and COL1α1 markers [176]. Particularly, it was shown that the polyphenol equol induced the proliferation of rat primary osteoblasts and increased ALP and OCN levels in this in vitro experimental model [177]. Notably, it was reported that not only fruits and vegetables but also cereals contain several polyphenols and other bioactives with osteogenic properties, like orthosilicic acid (H_2_SiO_4_) [178]. Interestingly, it was shown that the pro-mineralizing component (orthosilicic acid and vitamin K2) of the nutraceutical BlastiMin Complex^®^ (Mivell, Fano, Italy) in combination with the anti-inflammatory component of the formulation (curcumin, polydatin, and quercetin) acts synergistically in inducing the expression of the osteogenic marker *COL1A1* in young human BM-MSCs cells [179]. Furthermore, the combination of these five bioactive compounds can synergistically increase the expression levels of the osteogenic marker *ALP* in senescent human BM-MSCs and decrease the protein levels of the pro-inflammatory markers p-p38, p-NF-kB, Monocyte chemoattractant protein-1 (MCP-1), and Interleukin-8 (IL-8), demonstrating the in vitro pro-osteogenic and anti-inflammatory effects of the nutraceutical BlastiMin Complex^®^ (Mivell, Fano, Italy) [5].

## 3. Delivery Strategies to Improve Polyphenol Bioavailability

Many phytochemicals are characterized by a high molecular weight, poor aqueous solubility, limited gastrointestinal permeability, extensive pre-systemic metabolism, poor stability in the harsh gastrointestinal milieu, a short half-life, and non-specific distribution to organs. Therefore, innovative oral delivery systems to improve phytochemical bioavailability have been designed [180,181,182,183,184]. Conventional drug delivery systems like microspheres [185], microemulsions [186], amorphous solid dispersion [187], β-cyclodextrin [188], and pH-sensitive hydrogels [189,190] have been used to deliver polyphenols and other phytochemicals for the treatment of several pathologies [191]. Notably, nanomedicine can effectively improve the oral delivery efficacy of natural compounds, such as polyphenols, by circumventing various delivery restrictions [192,193]. In parallel, the rapidly growing application of nanotechnology to nutraceuticals, particularly through nano prebiotics and nano probiotics, underscores a transformative shift in enhancing the bioavailability of active ingredients. This innovative approach, involving the encapsulation of prebiotics, probiotics, and synbiotics in nanoparticles for improved absorption in the gastrointestinal tract, heralds a new era in therapeutic and nutraceutical outcomes. The development of nanofibers for probiotic delivery and synbiotic-based nanoparticles represents cutting-edge trends in this domain, although the field is still in its infancy, with limited experimental studies underscoring the necessity for further research on their effectiveness, bioavailability, and safety [194]. Notably, oral nano delivery systems can protect natural molecules from degradation in the gastrointestinal tract, are able to improve the pharmacokinetic and pharmacodynamic characteristics of the phytochemicals, and can also guarantee the delivery of the bioactives to the target organs and sustained drug release [181]. The different types of oral nano delivery systems reported for phytochemicals are: polymeric nanoparticles (chitosan-based, alginate/chitosan-based, gum-based, gum/chitosan-based, dextran-based, PLGA-based, PLA-Based, PCL-based, PVA-based), lipid-based nano systems (nanoemulsions, self-nanoemulsifying drug delivery systems, solid lipid nanoparticles, nanostructured lipid carriers), vesicular systems (liposomes, nanocochelates, niosomes, phytosomes), micelles, inorganic nanocarriers (polymeric and metallic nanoparticles, carbon nanotubes, mesoporous silica nanoparticles), and nanosuspensions [181,195]. Interestingly, a bottom-up ionic gelation method was applied to prepare chitosan nanoparticles encapsulating ferulic acid [196] or curcumin [197]. It was reported in L6 rat skeletal muscle cells that curcumin chitosan nanoparticles exhibited a superior effect on the translocation of GLUT4 to the cell surface as compared to free curcumin [197]. In another study, ferulic acid chitosan nanoparticles showed 4-fold enhanced oral bioavailability in vivo compared with free ferulic acid and displayed better antidiabetic potential in streptozotocin-induced diabetic rats [196]. Notably, it was shown that the alginate-coated chitosan core-shell nanocarrier system was able to effectively deliver naringenin to streptozotocin-induced diabetic rats, increasing the bioavailability of this phytochemical [198]. Similarly, in another study, pH-sensitive polymeric nanoparticles with a core-shell-corona morphology for encapsulating quercetin were prepared using succinyl chitosan and alginate [199]. Compared with native naringenin and quercetin, both core-shell nanoparticles exerted increased hypoglycemic effects and an effective maintenance of glucose homeostasis in streptozotocin (STZ)-induced diabetic rats with no toxicity in vivo [198,199]. Notably, it was reported that glycyrrhizin-loaded nanoparticles based on gum arabica and chitosan exerted striking antihyperglycemic and antihyperlipidemic effects in type 2 diabetic rats [200]. Interestingly, in an in vitro study on primary hepatocytes, berberine-loaded O-hexadecyl-dextran nanoparticles were very effective in preventing high glucose-induced oxidative stress, mitochondrial depolarization, and downstream events of apoptotic cell death [201]. Particularly, it was shown that nanoemulsions enhanced the oral bioavailability of berberine in vivo by 212% and reduced the blood glucose levels of diabetic mice by 3-fold [202]. Interestingly, the anthocyanidin pelargonidin was encapsulated in PLGA to obtain nano-pelargonidin, which protected hyperglycemic LG cells against the destruction of mitochondrial membranes, DNA damage, and oxidative stress [203]. Furthermore, pelargonidin exerted ant-diabetic effects and showed a 10-fold greater protective effect than native pelargonidin with an equivalent dose [203]. Notably, fisetin was efficiently encapsulated in PLGA-PEG-COOH nanoparticles, which could preserve and protect the release of fisetin under gastric-stimulated conditions, along with controlling its release in the intestinal medium and increasing the antioxidant and α-glucosidase inhibition activities of this polyphenol [204]. Notably, resveratrol was loaded into a self-nanoemulsifying drug delivery system, and this resveratrol nanoformulation displayed significant hypolipidemic, hypoglycemic, and neuroprotective effects on STZ and glucose-induced diabetic rats [205,206]. In another study, resveratrol was encapsulated in nanoliposomal formulations, which significantly decreased glucose levels and increased insulin levels in streptozotocin-induced diabetic β-TC3 cells and exerted increased antioxidant activity as compared with native resveratrol [207]. Interestingly, solid lipid nanoparticles of myricitrin exhibited antioxidant, antidiabetic, and antiapoptotic activities in STZ-induced diabetic mice [100]. In another study, baicalin-loaded nanostructured lipid carriers showed good physical stability and had better hypoglycemic and hypolipidemic effects in vivo when compared with pure baicalin [208]. Notably, embelin-loaded niosomes remarkably decreased lipid peroxidation, increased reduced glutathione (GSH), CAT, and SOD levels, and exerted antidiabetic effects in an in vivo experimental model [71]. Notably, the in vivo oral bioavailability of amentoflavone-loaded micelles was 3.2 times higher than that of native amentoflavone, and this nanoformulation exerted a remarkable anti-diabetic effect in insulin-resistant diabetic mice [209]. In conclusion, oral nano drug delivery systems used to improve the bioavailability of phytochemicals have the following advantages: (1) bioactive-encapsulated nano delivery systems can improve the stability of the natural molecules and protect them from enzymatic and/or chemical degradation in the gastrointestinal tract; (2) nano delivery systems increase the cellular drug uptake or block drug efflux mechanisms, further improving the pharmacokinetic and pharmacodynamic profile of natural compounds; (3) the nano delivery systems enhance the biological effects of phytochemicals, increase the bioavailability of the natural molecules, reduce the dose required to exert the investigated biological effects and allow the targeting of a specific therapeutic site to counteract the development of several pathological conditions [181]. The molecular targets modulated by the polyphenols described in this review in both in vitro and in vivo experimental models are reported in Table 2, which also shows the biological activities of these natural molecules associated with preventive and therapeutic effects against T2DM and osteoporosis pathologies.

Highlights: The nano delivery technologies used by researchers to increase the bioavailability of many polyphenols led to an improvement in the anti-diabetic effects of ferulic acid, curcumin, naringenin, quercetin, glycyrrhizin, berberine, pelargonidin, fisetin, resveratrol, myricitrin, baicalin, embelin, and amentoflavone. Notably, the reported inhibition of insulin resistance mechanisms and the decrease in blood glucose levels were also determined by an increase in the antioxidant activities of berberine, resveratrol, myricitrin, fisetin, pelargonidin, and embelin. Notably, the most interesting results were obtained with the improvement in the anti-apoptotic effects of berberine, resveratrol, and myricitrin, which were able to block the depolarization of the mitochondrial membrane and inhibit the intrinsic pathway of apoptosis that determined the death of pancreatic cells in both in vitro and in vivo experimental models.

## 4. Polyphenols Used for Clinical Management of Type 2 Diabetes Mellitus and Osteoporosis

### 4.1. Type 2 Diabetes Mellitus Clinical Trials

Interestingly, recent meta-analyses of prospective cohort studies have confirmed that the intake of polyphenol-rich foods is associated with a lower risk of T2DM development and an improvement in the health conditions of diabetic patients [210,211]. Notably, in a small meta-analysis, in three out of five randomized clinical trials, it stated a reduction in fasting blood glucose (FBG) and HbA1c using doses between 250 and 1000 mg of curcumin and after treatments between 10 days and 9 months [212]. In another study (9 months, 120 patients treated and 120 controls), curcuminoids-treated prediabetic subjects (1.5 g/day) did not develop T2DM, meanwhile, in the placebo group, 16% of patients developed T2DM [213]. Interestingly, the treated group also showed lower CRP levels and improvements in pancreatic β cell function. Moreover, it was reported that in T2DM patients (50 treated and 50 controls), curcuminoids (500 mg/day for 3 months) decreased FBG, CRP, and HbA1c levels [214]. Regarding the stilbene resveratrol, a clinical trial composed of a small sample (total of 19 patients, 10 intervened, and 9 controls) found that 10 mg/day of resveratrol-loaded capsules administered for a month improved homeostasis model assessment for insulin resistance (HOMA-IR) [215]; whereas in another clinical trial performed in 29 control subjects and 28 T2DM subjects treated with 250 mg/day of resveratrol for 3 months, there was a reduction in HbA1c levels [216]. Notably, in a clinical trial with 97 elderly patients with T2DM, it was shown that resveratrol treatment reduced HbA1c levels, CRP, and lipoperoxide values [217], while a combined treatment with resveratrol and delta-tocotrienol in 82 patients with metabolic syndrome reduced fasting plasma glucose, CRP, IL-6, and TNF-α levels [218]. Particularly, in a clinical trial with 50 T2DM patients treated with rutin, there was a decrease in IL-6 and HbA1c levels and an increase in total antioxidant capacity [219], while in a dietary intervention among 31 patients with T2DM, a 6-week daily intake of 500 mg of hesperetin significantly decreased the levels of the inflammatory markers CRP and TNF-α and increased the serum total antioxidant capacity [220]. Interestingly, epigallocatechin-3-gallate (EGCG) supplementation at a dosage of 300 mg/day for 8 weeks significantly decreased the level of FBG, CRP levels, and body mass index compared to the baseline among 50 patients with T2DM [221].

Highlights: The polyphenols curcumin, resveratrol, rutin, and EGCG all exerted anti-diabetic effects in T2DM patients, and this biological activity was associated with the anti-inflammatory effects of these phytochemicals. Notably, the improvement of the patient’s health condition was also determined by the antioxidant properties of rutin and hesperetin, which were able to reduce the levels of the pro-inflammatory markers CRP and TNF-α, which are also involved in the induction of the apoptotic process of pancreatic cells.

### 4.2. Osteoporosis Clinical Trials

It has been shown that an increased osteoporosis risk during menopause is associated with a reduction in estrogen levels. Since many isoflavones are estrogen receptor-β (ER-β) agonists, they can inhibit osteoporosis development and induce the formation of new bone tissue [222]. Notably, it has been reported in several clinical trials that genistein administration (54 mg/day for 1 or 2 years) was able to prevent bone loss in postmenopausal women by significantly increasing LS BMD and FN BMD values, inducing an increase in the pro-osteogenic markers ALP and OCN, and reducing the levels of the bone resorption markers deoxypyridinoline (DPD) and pyridinoline (PD) [223,224]. Notably, other clinical studies have shown an augmentation of BMD values and the induction of pro-osteogenic markers in patients after treatment with genistein and other phytoestrogens [225,226]. Interestingly, the 24-month randomized, double-blind, placebo-controlled, two-period crossover trial RESHAW was conducted to evaluate the effects of resveratrol (75 mg twice daily) on bone health in postmenopausal women. The data obtained after 12 months of resveratrol supplementation (versus placebo) showed an increase in both the lumbar spine and neck of the femur BMD and a reduction in the levels of the bone resorption marker CTX [227]. Notably, a clinical trial that enrolled sixty healthy postmenopausal women, randomly assigned to receive 200 mg of fermented soy containing 25 mg of resveratrol and 10 mg of equol or a placebo for 12 months, showed that at the end of treatment, OCN, ALP, and BMD values significantly increased, and there was also a decrease in DPD in patients who had received a combination of resveratrol and equol compared to the placebo group [228].

Highlights: The polyphenols genistein, resveratrol, and equol prevented bone loss in patients by exerting pro-osteogenic effects and also reducing bone resorption, indicating that restoring the balance between the activity of osteoblasts and osteoclasts is extremely important to counteract the detrimental effects of osteoporosis.

### 4.3. Disadvantages Associated with the Use of Polyphenols

In vitro and in vivo studies have evidenced some adverse effects of polyphenols that must be taken into account before adding them to a diet for therapeutic purposes [229]. Notably, iron malabsorption effects, neuropathy, genotoxic effects, a decrease in the levels of thyroid hormones in plasma, and a reduction in the bioavailability of some synthetic drugs have been reported upon the administration of extremely high concentrations of polyphenols, indicating that a careful evaluation of polyphenol use regarding their purity, concentration, bioavailability, and doses is required [229].

## 5. Conclusions

**Figure 3:**
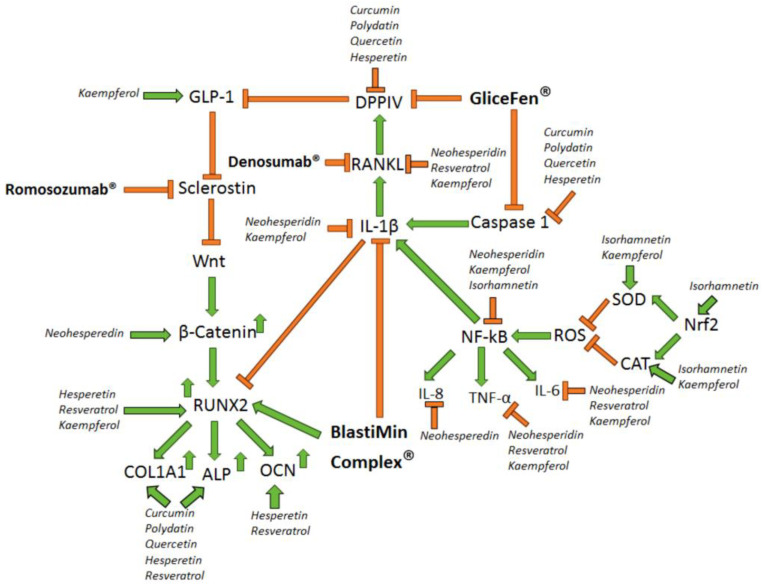
Interconnected molecular pathways that are involved in the development of T2DM, osteoporosis, oxidative stress, and chronic inflammatory state are shown. The effects of representative polyphenols on the modulation of the levels of specific molecular markers are also reported.

Human pathological conditions such as T2DM and osteoporosis are characterized by oxidative stress and a chronic inflammatory state, which lead to the worsening of the health conditions of patients affected by these diseases (Figure 3). Notably, the consumption of nutraceuticals containing different antioxidant and anti-inflammatory polyphenols could be an adjuvant supplement to counteract the hyperglycemia condition of T2DM and to reduce the levels of specific pro-inflammatory interleukins, such as IL-1β, which is activated by Caspase 1 in the inflammasome complex and contributes to the development of osteoporosis pathology and also to endothelium damage in T2DM (Figure 3) [3,230]. Interestingly, it has been reported that the molecular mechanisms that lead to T2DM and osteoporosis development are interconnected. In fact, it has been shown that RANKL, the molecule able to induce osteoclast differentiation, can also increase the levels and activity of the hyperglycemic enzyme DPPIV [231], which is able to cleave the hormone GLP-1, leading to insulin resistance development and hyperglycemia (Figure 3). In turn, GLP-1 can counteract the sclerostin-mediated inhibition of the Wnt/β-catenin pathway [232], leading to activation of Runx2, an increase in *COL1A1, ALP*, and *OCN* expression levels, and the differentiation of BM-MSCs into osteoblasts (Figure 3). Notably, the monoclonal antibodies Denosumab and Romosozumab have been developed to inhibit RANKL [231] and sclerostin, respectively [233], indicating the pivotal role of these molecular targets in osteoporosis development. Notably, it has been shown, in human young and senescent BM-MSCs, that the nutraceutical BlastiMin Complex^®^ (Mivell, Fano, Italy) can induce the differentiation of human BM-MSCs into osteoblasts, increase RUNX2 levels, and decrease IL-1β levels, indicating the pro-osteogenic and anti-inflammatory effects of this nutraceutical [5]. Since IL-1β can increase the levels of RANKL [230], which is able to increase DPPIV levels [231], we hypothesize that the biological effects exerted by the BlastiMin Complex^®^ could also counteract the detrimental effects of T2DM (Figure 3).

Interestingly, it has been reported that the nutraceutical GliceFen^®^ (Mivell, Fano, Italy), in human hepatocytes, can inhibit the catalytic activity of both the pro-inflammatory enzyme Caspase 1 and the hyperglycemic enzyme DPPIV [4]. As it was shown that the inhibition of DPPIV activity leads to an increase in GLP-1 levels and that this hormone can inhibit sclerostin and activate the Wnt/β-catenin/RUNX2 molecular pathway [232], we hypothesize that the biological effects exerted by the nutraceutical GliceFen^®^ could also counteract the detrimental effects of osteoporosis (Figure 3). Notably, it has been shown that T2DM and osteoporosis pathologies are so connected that diabetic osteoporosis has been increasingly recognized as an important complication of diabetes, and researchers have started to investigate the anti-diabetic and anti-osteoporotic effects of several bioactives, including polyphenols [234]. Notably, it has been shown that the polyphenol icariin, in an in vivo experimental model of diabetic osteoporosis, reduced blood glucose levels, increased the BMD of diabetic rats, and decreased the levels of the bone resorption markers CTX and TRACP 5b, indicating that this polyphenol can target a molecular network that regulates the development of both T2DM and osteoporosis [234]. Like icariin, many other polyphenols can exert anti-diabetic and pro-osteogenic effects concurrently (Figure 3). In fact, curcumin, polydatin, quercetin, and hesperetin can increase the levels of the pro-osteogenic markers *COL1A1* and *ALP* and inhibit the catalytic activity of the enzymes caspase 1 and DPPIV, which play a fundamental role in the development of the insulin resistance condition in T2DM (Figure 3). Furthermore, kaempferol can increase the levels of GLP-1 to counteract the detrimental effects of T2DM and is able to increase the RUNX2 levels and the CAT and SOD activity to exert pro-osteogenic effects and antioxidant effects, respectively, while neohesperidin can reduce the levels of the pro-inflammatory markers IL-8, TNF-α, IL-6, and IL-1β and concurrently increase the levels of β-catenin to exert both anti-inflammatory and pro-osteogenic effects (Figure 3). This scientific evidence indicates that the anti-diabetic and anti-osteoporotic effects of the individual polyphenols could benefit from synergistic effects associated with a nutraceutical formulation based on their combination. The novelty of the in vitro experimental results obtained with the two nutraceuticals GliceFen^®^ and BlastiMin Complex^®^ is focused on the synergistic biological effects exerted by the combination of all the natural molecules present in these formulations, indicating that the combination of several polyphenols with pro-osteogenic, antidiabetic, antioxidant, and anti-inflammatory effects could be used as adjuvants in the future, in combination with synthetic drugs, to develop innovative therapeutic strategies for the treatment of T2DM and osteoporosis human pathologies [4,5]. Interestingly, Shah et al. recently published a review focused on dietary polyphenols targeting individual molecular pathways that lead to the development of several age-associated diseases, such as T2DM, osteoporosis, cancer, and neurodegenerative diseases [229]. Notably, these authors described the molecular pathways that have to be modulated for counteracting the detrimental effects of osteoporosis pathology, focusing on the role of β-catenin in osteoblast proliferation and bone formation and of RANKL in bone resorption, but the molecular markers (like RUNX2, OCN, ALP, COL1α1, and OPN) that lead to the differentiation of human BM-MSCs in osteoblasts should also be considered. In addition, the oxidative stress and the pro-inflammatory microenvironment in the bone tissue play a pivotal role in osteoporosis development and are interconnected with the imbalance of the bone resorption and bone formation mechanisms, indicating that the pro-oxidant mechanisms and the chronic inflammatory state must also be inhibited to develop an efficient therapeutic strategy against osteoporosis. Notably, the same authors described the molecular pathways that have to be modulated for counteracting the detrimental effects of T2DM, focusing on oxidative stress, ROS-dependent DNA damage, and the senescence of pancreatic cells, but the markers (such as GLP-1 and GLUT4) and the enzymes (such as DPPIV and caspase 1) involved in the development of insulin resistance mechanisms should also be considered. Moreover, the molecular pathways of chronic inflammation, which lead to insulin resistance, a decrease in insulin receptor levels, and apoptosis induction in pancreatic cells, must also be targeted to develop innovative therapeutic approaches for T2DM treatment. In addition, the different chemical structures of polyphenols and, in particular, flavonoids should be taken into account to select the most suitable bioactives to target specific pathological conditions. In fact, it has been reported that flavonoids benefit from the presence of the C2-C3 double bond and the hydroxyl groups of the C3′, C4′, C5, and C7 positions for exerting antioxidant, anti-inflammatory, and anti-diabetic effects concurrently. Considering all the above-described notions, the novelty of this review is represented by the concept that an innovative therapeutic approach based on the use of polyphenols must take into account that the molecular pathways of oxidative stress, chronic inflammatory state, osteoporosis, and insulin resistance are all interconnected in a network of molecular markers that interact with each other, and, consequently, targeting only one of these molecular pathways (such as the old concept of focusing solely on pathophysiological mechanisms of oxidative stress) represents an ineffective therapeutic strategy. The use of nutraceutical formulations based on different polyphenols that can concurrently inhibit the chronic inflammatory state, oxidative stress, and insulin resistance mechanisms (GliceFen^®^ by Mivell, Fano, Italy) [3,4] or can concurrently inhibit oxidative stress and the chronic inflammatory state and also restore the balance between bone resorption mechanisms and new bone formation, inducing pro-osteogenic effects and the differentiation of human BM-MSCs into osteoblasts (BlastiMin Complex^®^ by Mivell, Fano, Italy) [3,5] represents an innovative concept for developing effective therapeutic strategies that can prevent and counteract the detrimental effects of several human chronic diseases, like T2DM and osteoporosis. Starting with this concept, the use of a selected formulation of polyphenols in combination with conventional anti-diabetic and anti-osteoporosis synthetic drugs should be explored to investigate the beneficial effects of these innovative therapeutic approaches. Furthermore, the study of the ability of polyphenols to modulate the interaction of osteoblasts and osteoclasts within the bone tissue microenvironment and to regulate the interaction between the pancreas and liver regarding the development of multiorgan insulin resistance conditions will pave the way for a deeper understanding of the molecular pathways that play a role in the development of the pathological conditions of osteoporosis and T2DM and for identifying new molecular biomarkers for the treatment of these human chronic diseases.

## Figures and Tables

**Table 2:** Antioxidant, anti-inflammatory, anti-diabetic, and pro-osteogenic activities of polyphenols reported in this Review. The in vitro/in vivo experimental models used for the studies and the molecular targets modulated by the described polyphenols are also indicated.

Polyphenols	Study Type	Biological Activities	Outcomes of Studies	References
Isorhamnetin	In vitro; In vivo	Antioxidant; Anti-inflammatory; Anti-diabetic	Increase: GLUT4; SOD; CAT; Nrf2 Decrease: NF-kB	[97]
Apigenin	In vitro; In vivo	Antioxidant; Anti-inflammatory; Anti-diabetic	Increase: SOD; CAT Decrease: α-glucosidase; NF-kB	[22,98]
Licochalcone A	In vivo	Anti-diabetic	Increase: IRS-2/PI3K/Akt	[42]
Myricitrin	In vitro; In vivo	Anti-diabetic; Antioxidant; Anti-inflammatory	Increase: HO-1; NQO1; GLUT4 Decrease: NF-kB	[43,99,100]
Biochanin A	In vivo	Antioxidant; Anti-inflammatory; Anti-diabetic	Increase: SIRT1 Decrease: HbA1c; NF-kB	[101]
Formononetin	In vivo	Antioxidant; Anti-diabetic; Pro-osteogenic	Increase: SIRT1; RUNX2; OCN; *COL1A1*; OPN Decrease: HbA1c	[102,159,160]
Hesperetin	In vitro; In vivo	Antioxidant; Anti-inflammatory; Anti-diabetic; Pro-osteogenic	Increase: INSR; SIRT1; ALP; RUNX2; OCN; *COL1A1* Decrease: Semaphorin 3E; DPPIV; Caspase 1	[4,46,175,176]
Naringenin	In vitro; In vivo	Anti-inflammatory; Anti-diabetic; Antioxidant	Increase: INSR; SIRT1; GLUT4; SOD; CAT Decrease: Semaphorin 3E; DPPIV; TNF-α; Caspase 1	[4,104,188,198]
Kaempferol	In vitro; In vivo	Anti-inflammatory; Anti-diabetic; Pro-osteogenic; Antioxidant	Increase: SOD; CAT; GLP-1; ALP; RUNX2 Decrease: IL-1β; NF-kB; IL-6; TNF-α; NFATc1; RANKL	[105,106,107,108,168,169]
Epigallocatechin-3-gallate	In vivo	Antioxidant; Anti-diabetic; Anti-inflammatory	Increase: SOD, CAT Decrease: HbA1c; IL-1β	[109]
Galangin	In vitro	Anti-diabetic	Decrease: DPPIV	[110]
Fisetin	In vitro; In vivo	Anti-diabetic; Pro-osteogenic	Increase: RUNX2; *COL1A1*; OCN; OPG Decrease: HbA1c; α-glucosidase	[112,113,155,204]
Myricetin	In vivo	Anti-diabetic; Antioxidant	Increase: GLP-1; CAT; SOD Decrease: DPPIV	[114]
Cyanidin-3-O-glucoside	In vitro; In vivo	Antioxidant; Anti-inflammatory; Anti-diabetic	Increase: GLUT4; SOD Decrease: DPPIV; α-glucosidase; TNF-α	[115,116]
Vaccarin	In vitro; In vivo	Antioxidant; Anti-diabetic	Increase: GPx Decrease: ROS/AMPK/miRNA-34a/eNOS	[54,117]
Taxifolin	In vitro; In vivo	Antioxidant; Anti-diabetic	Increase: SOD; CAT; GPx Decrease: α-amylase	[118,119]
Curcumin	In vitro	Anti-inflammatory; Anti-diabetic; Pro-osteogenic	Increase: INSR; GLUT4; *ALP*; *COL1A1* Decrease: Semaphorin 3E; DPPIV; Caspase 1	[4,5,197]
Polydatin	In vitro	Anti-inflammatory; Anti-diabetic; Pro-osteogenic	Increase: INSR; *ALP*; *COL1A1* Decrease: Semaphorin3E; DPPIV; p-P38; Caspase 1	[4,5,56]
Quercetin	In vitro; In vivo	Anti-inflammatory; Anti-diabetic; Pro-osteogenic; Antioxidant	Increase: INSR; *ALP*; *COL1A1* Decrease: Semaphorin 3E; DPPIV; ROS; Caspase 1	[4,5,185,199]
Quercetin-3-O-β-D-galactopyranoside	In vitro	Pro-osteogenic	Increase: ALP; Runx2; OCN	[156]
Vanillic acid	In vivo	Pro-osteogenic; Anti-inflammatory	Increase: ALP; OCN Decrease: IL-1β; IL-6; TNF-α	[59]
Ugonin K	In vitro	Antioxidant; Pro-osteogenic	Increase: ALP; OCN; Runx2 Decrease: ROS	[60,157]
Neobavaisoflavone	In vitro	Pro-osteogenic	Increase: ALP; *COL1A1*; OCN	[61]
Salvianolic acid B	In vitro; In vivo	Pro-osteogenic	Increase: Runx2; ALP; *COL1A1* Decrease: TRACP-5b	[62,158]
Kobophenol A	In vitro	Antioxidant; Pro-osteogenic	Increase: ALP Decrease: ROS	[63]
Genistein	In vivo	Anti-inflammatory; Pro-osteogenic	Increase: OPG; ALP; Runx2 Decrease: RANKL; NF-kB	[161,162,163]
Resveratrol	In vitro; In vivo	Antioxidant; Anti-diabetic; Pro-osteogenic; Anti-inflammatory	Increase: SIRT1; p-AMPK; Col1α1; Runx2; OCN; ALP Decrease: TNF-α; IL-6; RANKL; TRAP-5b	[142,164,205,206,207]
Puerarin	In vivo	Pro-osteogenic	Increase: OPG; OPN Decrease: RANKL	[165]
Icariin	In vitro; In vivo	Pro-osteogenic	Increase: cAMP/PKA/CREB	[65]
Tanshinol	In vivo	Pro-osteogenic	Increase: β-catenin/Runx2	[66]
Naringin	In vitro; In vivo	Antioxidant; Anti-inflammatory; Pro-osteogenic	Increase: ALP; OCN Decrease: RANKL; NF-kB; ROS	[166,167]
Poncirin	In vitro; In vivo	Pro-osteogenic	Increase: OCN; Runx2; ALP Decrease: CTX	[67]
Rutin	In vitro; In vivo	Anti-inflammatory; Pro-osteogenic	Increase: ALP; OCN Decrease: IL-6; TNF-α	[148,170]
Neohesperidin	In vitro; In vivo	Antioxidant; Anti-inflammatory; Pro-osteogenic	Increase: Runx2; OCN; ALP; β-catenin Decrease: RANKL; Cathepsin K; IL-1β; IL-6; IL-8; TNF-α; NF-kB; NFATc1	[46,171,172,173]
Hesperidin	In vitro; In vivo	Antioxidant; Anti-inflammatory; Pro-osteogenic	Increase: SOD; GPx; Runx2; ALP; OCN Decrease: IL-1β; IL-6; TNF-α	[46,103,174]
Baicalin	In vivo	Anti-diabetic	Decrease: HbA1c	[208]
Ferulic acid	In vivo	Anti-diabetic	Decrease: Blood glucose	[196]
Glycyrrhizin	In vivo	Anti-diabetic	Decrease: HbA1c	[200]
Berberine	In vivo	Antioxidant; Anti-diabetic	Increase: SOD Decrease: Blood glucose; ROS	[201,202]
Pelargonidin	In vitro	Antioxidant; Anti-diabetic	Increase: GLUT4 Decrease: ROS	[203]
Embelin	In vivo	Antioxidant; Anti-diabetic	Increase: SOD; CAT Decrease: Blood glucose	[71]
Amentoflavone	In vivo	Anti-diabetic	Increase: PI3K/Akt	[209]
Phloridzin	In vitro	Anti-diabetic	Increase: p-AMPK; p-Akt	[120]
Equol	In vitro	Pro-osteogenic	Increase: OCN; ALP	[177]

## Data Availability

No new data were created or analyzed in this study. Data sharing is not applicable in the review article.
